# Noise Reduction in Human Motion-Captured Signals for Computer Animation based on B-Spline Filtering

**DOI:** 10.3390/s22124629

**Published:** 2022-06-19

**Authors:** Mehdi Memar Ardestani, Hong Yan

**Affiliations:** 1 Center for Intelligent Multidimensional Data Analysis, Hong Kong Science Park, Shatin, Hong Kong; h.yan@cityu.edu.hk; 2 Department of Electrical Engineering, City University of Hong Kong, Kowloon, Hong Kong

**Keywords:** motion capture, motion data processing, digital signal processing, motion smoothing, motion denoising, B-splines

## Abstract

Motion capturing is used to record the natural movements of humans for a particular task. The motions recorded are extensively used to produce animation characters with natural movements and for virtual reality (VR) devices. The raw captured motion signals, however, contain noises introduced during the capturing process. Therefore, the signals should be effectively processed before they can be applied to animation characters. In this study, we analyzed several common methods used for smoothing signals. The smoothed signals were then compared based on the smoothness metrics defined. It was concluded that the filtering based on the B-spline-based least square method could achieve high-quality outputs with predetermined continuity and minimal parameter adjustments for a variety of motion signals.

## 1. Introduction

Motion capture (MoCap) has become one of the main sources of simulating real, natural motions. The produced motions have extensive applications in motion analysis research, animation, gaming, and virtual reality products.

The raw motion signals captured, however, are usually contaminated with noises from different sources. Therefore, these signals should undergo special processes to generate high-quality motion signals that can be used in the various applications mentioned above. Moreover, due to the rapid development of virtual reality (VR), augmented reality (AR), extended reality (XR), mixed reality (MR) and metaverse-based technologies, there is an increased need to process human motion-captured signals in computer animation. For these reasons, studies of motion smoothing or denoising techniques for generating more realistic motion signals have always been appealing and can gain useful applications in real-world digital entertainment systems.

Recently, a systematic review of the applications of motion capture (MoCap) in various industries was studied by Menolotto et al. [1]. Their study mainly focused on applications in the construction, robotics, and automotive industries. Motion capturing is achieved by using various motion-capture devices, including body-fixed sensors [2], monocular cameras [3], marker [4] or marker-less [5] approaches, and visual information [6], that can track certain body points of the moving target object. Moreover, using infrared sensors, depth cameras such as Microsoft Kinect and Vicon 3D [7] provide a convenient way to track and extract motion data from depth maps with high accuracy [8,9,10]. Furthermore, Chatzitofis et al. [11] proposed DeepMoCap, a marker-based, optical motion-capture method equipped with a multistage fully convolutional network (FCN) deep-learning architecture. The proposed method could achieve up to 4.5% more accuracy compared to the next best model in their study. For the design aspect of motion-capture wearables, readers are referred to a study by Marin et al. [12] in which they presented a design framework that could help identify the design requirements for creating wearable products.

Regarding the smoothing of data obtained from a continuous experiment, vast literature is available. One of the earliest works was performed by Savitzky and Golay [13] in which they leveraged the power of early computers to compute the smooth curve that best fit the data using a simplified least square method. Eilers and Marx [14] combined B-spline and difference penalties to form the so-called P-splines. They also presented a way to calculate the optimal value of the smoothness penalty factor using the Akaike information criterion (AIC). Moreover, in 2003, Eilers [15] proposed an improved version of the Savitzky–Golay filter, the so-called perfect smoother, using a discrete penalized least square method that offered continuous control over smoothness of the curve. Moreover, this method allowed for fast leave-one-out cross-validation.

Hsieh [16] utilized B-spline wavelets to smooth the motion data. In his work, the noise was modeled as high-frequency, small-amplitude components added to the original signal, and then the noisy signal was decomposed using a wavelet transform process to detect and eliminate the noise.

Qi et al. [17] presented and compared three different filtering methods, namely wavelet filter, Gauss filter, and mean filter, to produce natural motion data. They smoothed the position and orientation signals separately.

Lou and Chai [18] presented an example-based approach. They formulated the signal denoising process as a nonlinear optimization process in which the objective function had two parts to ensure the minimum distance between the noisy input and the filtered signal, as well as the preservation of the spatial–temporal patterns of the human motion signals.

Using image and signal processing techniques such as multiresolution motion filtering, multitarget motion interpolation with time-warping, and motion displacement mapping, Bruderlin and Williams [19] used existing motion data to design and modify the animated motions. Their method of blending motions could be used independently of the way the animation was produced, whether it was achieved via traditional keyframing, motion capture, or procedural animation.

In the case of motion signals, the signals usually contain a wide range of frequencies, depending on the activity being captured and the channel being processed. Moreover, the signals may contain outliers, or some data may be missing, depending on the capturing tools, their accuracy, and the environment conditions.

There are several factors that contribute to the noise in the captured signal. They can broadly be categorized into internal and external factors. By internal factors, we mean noises associated with electronic parts used in the capturing device. Several contributing factors for external noises can be due to (1) the experiment environment condition related to the temperature, the weather, or the lighting; (2) the kind of activity being captured (for example if it involves sudden movements or sudden changes in direction); (3) accidental touching of the markers attached to the capture subject; and (4) interference by other radio frequency signals present in the lab environment.

As can be seen, most of the contributing factors are inevitable in the process of motion capturing. Therefore, denoising or other post-processing remedies, for example, in the case of missing data, are essential parts of the process to acquire high-quality, smooth motion signals.

The classical filters, such as moving average or Kalman filters, may not perform well since they are too dependent on the current or immediate neighboring data being processed. This motivates the application of B-spline smoothing to motion signals. B-spline smoothing is appealing to us because of its flexible nature, i.e., the piecewise connectivity between the curves and, more importantly, because the degree of continuity (and, hence, the smoothness) of the produced signals is guaranteed and can be readily increased with minimal effort. This is the unique property of B-spline-smoothed signals that cannot be accessed through other filters. Therefore, in cases of the presence of outliers or missing data, a B-spline smoother can naturally produce outputs that are reliable and smoothed to the required degree. Moreover, for studies involving the extraction of signal features such as velocity and acceleration for downstream data processing, such as clustering and classifier training [20], the ability to obtain reliable values for derivatives is required. B-spline smoothing can be used to denoise motion signals, which then can be used reliably for the accurate calculation of derivatives. This unique feature is not accessible using classical filtering methods such as moving average and Kalman filters. The limitation of the B-spline method, however, is that it can be used in an offline setting only. Nevertheless, since the applications of offline motion-captured signals are vast, the method can be useful in these applications. Furthermore, other filters, such as Kalman and moving average, can be used for online settings because their calculations are localized around the current timeframe. That is, they are based on the current datapoint and some immediate, neighboring, past datapoints, and they may not perform well when dealing with sudden local spikes in the signal. In addition, we use low-pass filters here since we are trying to eliminate shaky movements, which are mainly due to high-frequency noises, from our captured motion signals. Designing a specific band-pass filter for our workflow is not our intention in this study. Here, we aim for utilizing B-spline filtering because of the flexibility and the straightforward control it provides over the continuity of its piecewise connected curves.

In this paper, we compare three different smoothing methods and investigate their performances and limitations for the processing of motion signals. The methods we consider here are moving average smoothing, B-spline smoothing, and the well-known Kalman filter. The methods are introduced in Section 2. Their performances are subsequently analyzed through testing with simulated noisy signals and real captured motion signals in Section 3. The superior properties of B-spline-smoothed curves are also explained.

## 2. Materials and Methods

In this section, we present the formulations for three different motion-smoothing techniques, namely the moving average filter, B-spline smoothing, and the Kalman filter.

### 2.1. Moving Average Filtering

The moving average technique is one of the simplest yet most effective filters and is widely used in signal processing [13]. The idea is that the filtered value of the signal at the current time *t* is obtained by averaging the value of the signal in the current datapoint and a few other immediate, neighboring datapoints. The number of neighboring points contributing to the average value is adjusted by the user and can be varied based on the type and quality of the signals being processed. Moreover, the neighboring datapoints can be chosen from the past or future of the current datapoint at *t*. In the case of online filtering, only past datapoints are available. This method resembles convolution methods where the filtering process is conducted on a window of datapoints, and this window slides through all of the sampling datapoints. The values of the sampling points in each window can also be multiplied by a weighting coefficient. The averaging window can be chosen asymmetrically (i.e., only the past samples are used for averaging) or symmetrically (i.e., an equal number of points from the past and future of the current data point contribute to the averaging).

When the averaging window is asymmetric, we inevitably experience a phase-shift in the smoothed signal. This is because, at each point in time, considering the averaging window size of *n* datapoints, the value of the current datapoint is composed of (*n*−1)/*n* of the past data and only 1/*n* of the present data. For this reason, as well as for the sake of computational efficiency, the minimum number of sampling points that fulfill the desired level of smoothness of the signal should be chosen for moving average filtering, especially in the case of asymmetric averaging.

### 2.2. B-Spline Smoothing

B-splines have been used for the interpolation and approximation of data resulting from a variety of scientific experiments [14,21,22]. Their order of continuity (smoothness) can be readily increased. Since they are piecewise polynomials joined end-to-end, they offer great flexibility.

The B-spline approximation curve is written as:

(1) $$C=\sum_{i=1}^{m}N_{i,p}\left(\xi \right)P_{i},$$

where *P_i_* are the control points, *m* is the number of control points, and $N_{i,p}\left(\xi \right)$ are the B-spline basis functions of the degree *p* defined using the Cox–de Boor recursion formula as follows.

For *p* = 0:

(2) $$N_{i,0}\left(\xi \right)=\{\begin{array}{c} 1,\;\mathrm{if}\;\xi_{i}\leq \xi \leq \xi_{i+1},\\ 0,\;\mathrm{otherwise}. \end{array}$$

For *p* ≥ 1:

(3) $$N_{i,p}\left(\xi \right)=\frac{\xi -\xi_{i}}{\xi_{i+p}-\xi_{i}}N_{i,p-1}\left(\xi \right)+\frac{\xi_{i+p+1}-\xi }{\xi_{i+p+1}-\xi_{i+1}}N_{i+1,p-1}\left(\xi \right).$$

The control points *P_i_* are to be found. We used least square methodology to establish the optimal curve, which had the minimum distance error in the sense of the least square definition. That is:

(4) $$R=\overset{n}{\underset{j=1}{\sum }}{\left|y_{j}-C_{j}\right|}^{2}$$

where *C_j_,* which is the value of the B-spline curve *C* at point cinna corresponding to point *y_j_* in the signal, can be evaluated using Equation (1). Note that the number of control points could be less than the number of datapoints since we were approximating the signal curve. In the case of interpolation, the number of B-spline control points and the number of datapoints are equal. By differentiating Equation (4) with respect to *P_i_* and setting them to zero, the optimal curve could be found. We had *m* equations and *m* unknowns.

(5) $$\frac{\partial R}{\partial P_{i}}=\overset{n}{\underset{j=1}{\sum }}{\left|y_{j}-\overset{m}{\underset{i=1}{\sum }}N_{i,p}\left(\xi_{j}\right)P_{i}\right|}^{2}=0.$$

Since we were constructing the B-spline curve, the degree of smoothness of the curve was determined by the degree of B-spline basis functions. Furthermore, the flexibility of the curve could be adjusted by the number of control points (or, equally related, the number of knots in the knot vector).

### 2.3. Kalman Filter

The Kalman filter uses an iterative mathematical process, which is given a series of data inputs all containing noises, random errors, or uncertainty, and can quickly estimate the exact value of the signal being measured [23]. Kalman filtering is popular in applications such as the guidance and navigation of vehicles, aircraft, and robotic motion planning. Moreover, it has been used in processing signals when accurate estimates of measurements are needed.

The calculation of the value of measurement is performed in three stages, namely calculation of the Kalman gain, estimation of the current measurement, and estimation of the current error value, as follows:

(6) $$KG=\frac{E_{est}}{E_{est}+E_{mea}},$$

(7) $$EST_{t}=EST_{t-1}+KG\left(mea-EST_{t-1}\right),$$

(8) $$E_{est}=\left(1-KG\right)E_{est}.$$

Kalman filtering can be used in online, as well as offline, settings. However, the current gain and the current error estimate are adjusted based on the cumulative history of datapoints.

### 2.4. Research Method

**Figure 1:**
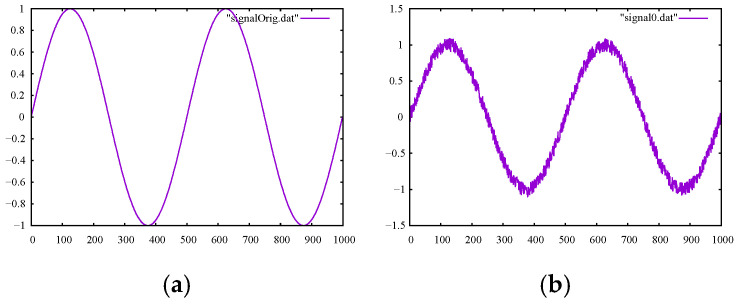
(**a**) The experimental true sine signal of frequency 1 and (**b**) the sine signal with randomly generated noise added.

To gain more insights about the methods, we considered two numerical case studies. For the first case study, we set up a sample sine signal, shown in Figure 1a, and added randomly generated white noise to the original signal, shown in Figure 1b.

For the second case study, we examined real-world captured motion data available from the Carnegie Mellon motion dataset [24] in more detail. Motion data in this database are captured using a Vicon motion-capture system with 12 infrared MX-40 cameras. The person carrying out the motions wears a black garment and 41 markers on different parts of their body. These markers are then detected by the infrared cameras, and their motions are recorded [24]. The methods discussed in the previous sections can be used for a variety of motion signals. To this end, the motion data we chose for the numerical experiments (subject #11, kick soccer ball), was from a character who, starting from a neutral pose, walked towards and then kicked a ball. Therefore, the motion of this kicking model included a variety of different movements, divided mainly into a simple walking motion and a more-involved kicking motion. For this motion data, there were 132 channels of motion signals. Each channel corresponded to the motion signal for one degree of freedom of a joint. For this motion data, the signals for the root-joint of the character, i.e., the hip movements, were considered. There were six channels related to this point, of which three were positional movements, and three were rotational movements. For our numerical experiments, we considered the signals from channels 1 and 3 of this joint. Channel 1 was selected since it mainly consisted of low-frequency components. Since smoothing filters are essentially low-pass filters, it is good to measure their ability to properly reproduce low-frequency inputs. The channel 3 signal was selected since it contained mid- to high-frequency components and, therefore, a fair number of opportunities for the filter to smooth out the noisy portion of the signal. For each case study, the chosen signals were then processed (smoothed) using the methods introduced in Section 2, namely the moving average, B-spline, and Kalman filters.

Several metrics were defined to measure the performance of the filtering methods. The SM1 parameter, as defined in Equation (9), was essentially the first-order backward difference formula in which the division by Δ*h*, the spacing between two consecutive frames, was omitted since the distance between the frames was constant and could be considered as one. SM1 can be understood as a measure of smoothness of the whole signal curve, or the sum of the slopes of the signal curve at consecutive points in time in the absolute sense (since only the magnitude of the slope was important for our measurement). A similar metric was previously used by [15] as a measure of the smoothness of the curve that then adjusted its effect using a penalty factor. SM1 was most meaningful when it was compared relatively for the signal curve before and after the filtering process. Therefore, the percentage of relative difference for this value (denoted by %SM1) between signals before and after filtering (denoted by SM1_0_ and SM1_1_, respectively) defined in Equation (10) is also presented in the corresponding tables. This can be interpreted as the reduction in high-frequency, shaky movements of the character (depending on what “high-frequency” means for that signal). When dealing with motion signals, we needed a measurable quantity that could indicate whether the output signal was a good fit for the original signal because there were a lot of channels to consider (~100 channels is very typical). %SM1 could serve this purpose. The threshold for the SM1 relative difference could be set for each channel separately. However, we observed in our numerical experiments that, as a rule of thumb, SM1 relative differences of around 10 to 15 percent gave acceptable outputs, while outputs with SM1 relative differences of more than 30 percent needed to be checked to ensure that the desired level of filtering was performed. The SM2 parameter defined in Equation (11) was known as the energy of the signal and was representative of the smoothness of the signal curve as a whole. The expression within the absolute symbols was essentially a first-order central difference formula with division by Δ*h* omitted because of the same reason discussed above in the explanation of Equation (9). Similar to the SM1 metric, the percentage of relative difference of SM2, denoted by %SM2 and defined in Equation (12), is also given in the corresponding tables. Parameters SM2_0_ and SM2_1_ in Equation (12) denote the values of the SM2 metric for signals before and after filtering, respectively.

(9) $$\mathrm{SM}1=\overset{n}{\underset{i=2}{\sum }}\left|y_{i}-y_{i-1}\right|$$

(10) $$\%\mathrm{SM}1=100\frac{\left(\mathrm{SM}1_{0}-\mathrm{SM}1_{1}\right)}{\mathrm{SM}1_{0}}$$

(11) $$\mathrm{SM}2=\overset{n-1}{\underset{i=2}{\sum }}{\left|y_{i-1}-2y_{i}+y_{i+1}\right|}^{2}$$

(12) $$\%\mathrm{SM}2=100\frac{\left(\mathrm{SM}2_{0}-\mathrm{SM}2_{1}\right)}{\mathrm{SM}2_{0}}$$

Furthermore, for all the figures in this paper, the curves corresponding to signals before and after filtering are respectively denoted by signal0_ and signal1_ prefixes in their legend boxes.

## 3. Results

In this section, following the numerical case studies set up in Section 2, we present and discuss the results obtained using the methods discussed in Section 2. In Section 3.1, we consider the simulated noisy sine signal as a benchmark experiment. We then continue with the same procedure for the real-life motion signal in Section 3.2.

### 3.1. Simulated Noisy Sine Signal

**Figure 2:**
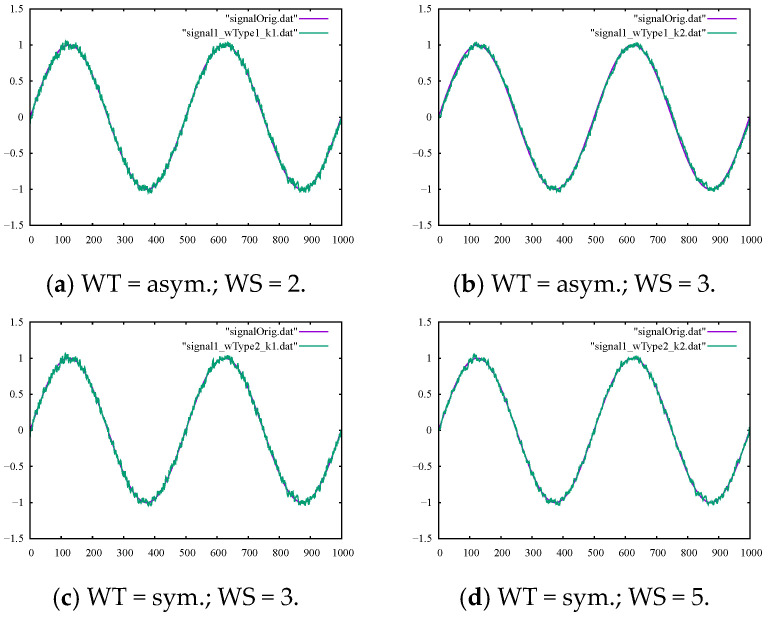
The output of the moving average filter with the ground-truth sine signal for comparison using (**a**) an asymmetric window of size 2, (**b**) an asymmetric window of size 3, (**c**) a symmetric window of size 3, and (**d**) a symmetric window of size 5.

We considered the noisy sine signal and denoised this signal using the methods discussed in Section 2. We started with the moving average filter to eliminate the noise, using windows of different types, i.e., symmetric or asymmetric, and sizes. By comparing the values for the SM1 and SM2 parameters presented in Table 1, it can be seen that the larger the averaging window size, the smoother the filtered signal. Moreover, the symmetric averaging windows tended to produce smoother curves compared to asymmetric windows. However, the outputs in Figure 2a–d show that filtered signals still contained high-frequency noises in the original signal. Therefore, the moving average filter was of limited effectiveness when the signal contained high-frequency noises of medium magnitude.

The performance of the B-spline method was largely determined by the number of control points used for approximation of the signal. As the number of control points was increased, the output became closer to the interpolation of datapoints in the signal. For approximation and smoothing purposes, only a fraction of the number of datapoints is usually used. This also helped with the performance of the method since the matrix produced during the procedure was reduced to a sparse matrix size of only 50 by 50. The number of control points is usually determined through numerical experiments depending on the number of datapoints, the characteristics of the signal, and the features of the signal to preserve.

**Figure 3:**
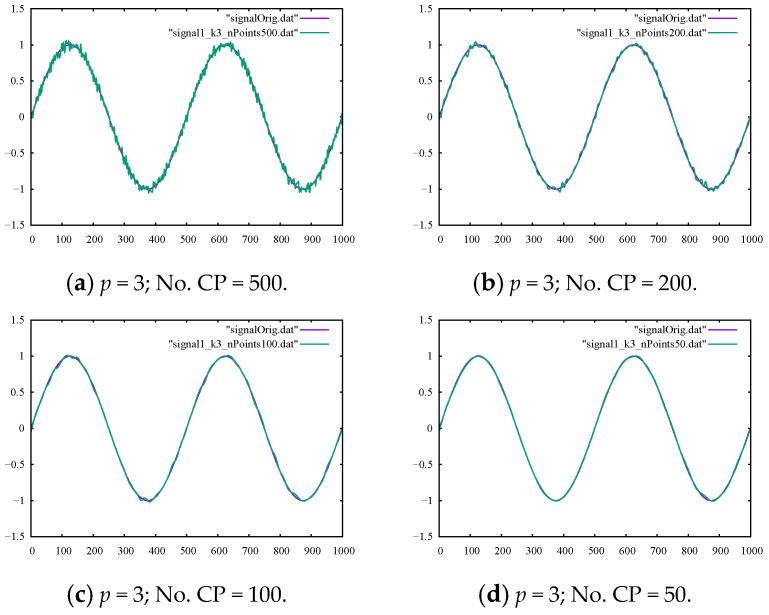
The output of the B-spline filter with the ground-truth sine signal for comparison using (**a**) 500, (**b**) 200, (**c**) 100, and (**d**) 50 control points.

Examining the metrics presented in Table 2, we can clearly see that we could achieve smoother signal curves by decreasing the number of control points used for creating the smooth curve. This trend could also be confirmed from the outputs given in Figure 3a–d.

**Figure 4:**
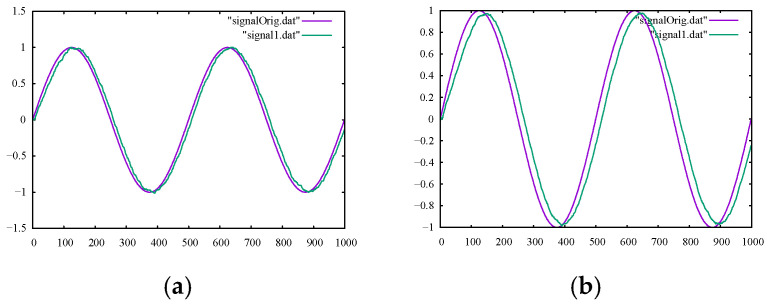
The output of the Kalman filter with the ground-truth sine signal for comparison using (**a**) $E_{mea}$ = 0.01 and (**b**) $E_{mea}$ = 0.02.

For the Kalman filter, the filtered output could be adjusted by tuning the $E_{mea}$ parameter, the measurement error. With higher values of measurement error, the Kalman filter tried to eliminate more noises and output slightly smoother signals, as can be attested by the smoothness metrics in Table 3. However, smoother signals came with more lags in the output, as plotted in Figure 4a,b.

Filtering the sine signal contaminated with artificial noises showed how capable various filtering methods were in controlled, lab-like experiments. As can be seen in Figure 2a–d, moving average filtering had very limited capability in effectively removing high-frequency noises in all the configurations. This was also true for the outputs of the Kalman filter shown in Figure 4a,b, while the B-spline filter could be readily adjusted to effectively remove the high-frequency noises from our true signal, as shown in Figure 3a–d. These numerical experiments, while simple, represented lab-like experiments with our filtering tools and set possible expectations when dealing with real-life motion signals, as explored in the next section.

### 3.2. Real Motion Signals

This section presents and discusses the results of the performance metric parameters introduced in Section 2.4 for the motion signals from channels 1 and 3 of the kicking character explained in Section 2.4.

**Figure 5:**
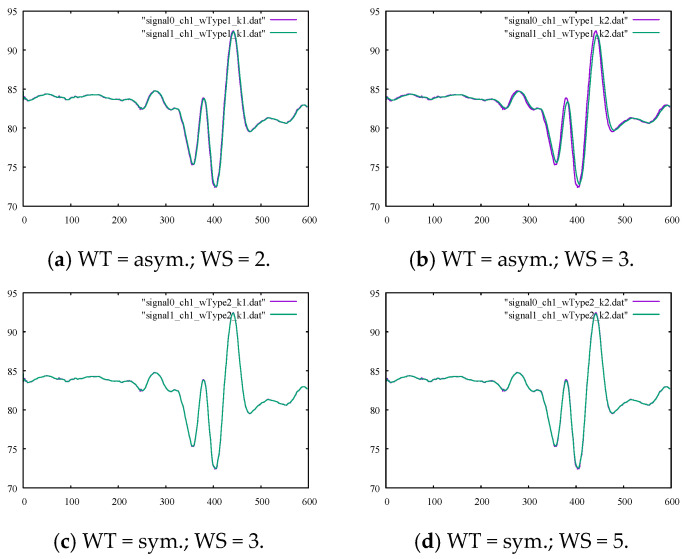
Original noisy motion signal from channel 1 of the root-joint of the kicking character versus the smoothed signal via the moving average filter using (**a**) an asymmetric window of size 2, (**b**) an asymmetric window of size 3, (**c**) a symmetric window of size 3, and (**d**) a symmetric window of size 5.

**Figure 6:**
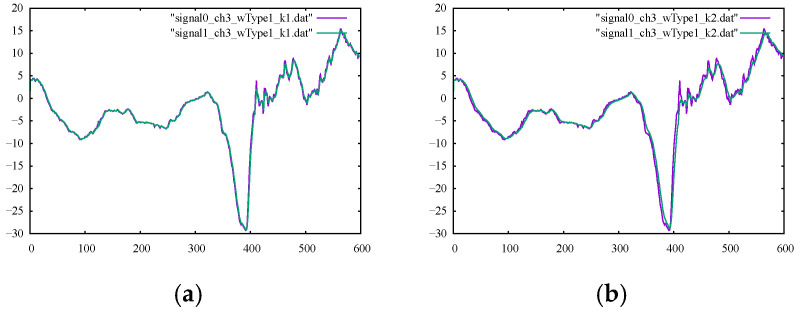

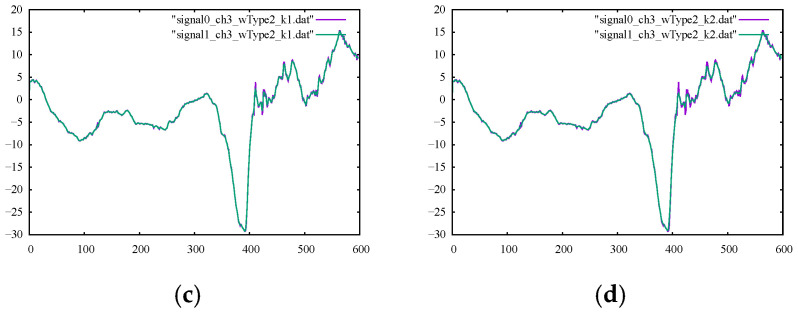
Original noisy motion signal from channel 3 of the root-joint of the kicking character versus the smoothed signal via the moving average filter using (**a**) an asymmetric window of size 2, (**b**) an asymmetric window of size 3, (**c**) a symmetric window of size 3, and (**d**) a symmetric window of size 5.

**Figure 7:**
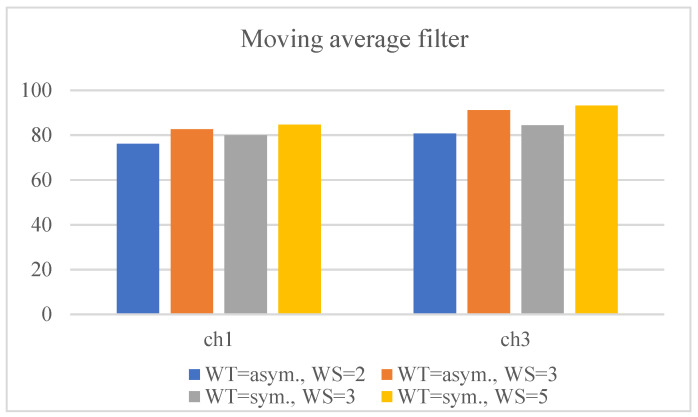
Percentages of relative difference for smoothness gained for the selected root-joint signals of the kicking character using the moving average filter with symmetric and asymmetric averaging windows of different sizes.

For the first numerical experiment, the moving average method was applied using symmetric and asymmetric averaging windows of different sizes. The results for the SM1 and SM2 metrics are presented in their corresponding columns of Table 4 and Table 5, respectively. The calculated percentages of the relative differences for SM1 and SM2 are listed in Table 6 and Table 7, respectively. The graphs of the outputs of the moving average filter using various window types and sizes with the original signals for channels 1 and 3 are drawn in Figure 5 and Figure 6, respectively. By examining the corresponding figures, it can be observed that, while the moving average filter performed well for signals with less high-frequency noises or movements (because of a highly localized averaging process), the filter was unable to omit high-frequency movements. To better interpret the results, the smoothness metrics in Table 7 are drawn in the bar chart in Figure 7. The trend was remarkably similar for both channels, i.e., the larger the averaging window, the smoother the output signals. Furthermore, symmetric windows could achieve higher smoothness values.

The performance metrics for the B-spline smoothing of the kicking model using 598, 200, 100, 50, and 25 control points are listed in their corresponding columns of Table 4 and Table 5. Their corresponding percentages of relative difference are given in Table 6 and Table 7. The results confirmed a clear gain in the smoothness metrics. The figures in Table 7 suggest that using a smaller number of control points led to an increase in the overall smoothness parameter of SM2 for both channels. Using fewer control points for smoothing the signal curves allowed for less strict compliance with the instantaneous signal values and, rather, provided more freedom to follow the global trend of the signal curve. However, higher smoothness did not always give the desired output since the output signal may be over-smoothed in a way that it no longer complied with the general trend of the original signal.

**Figure 8:**
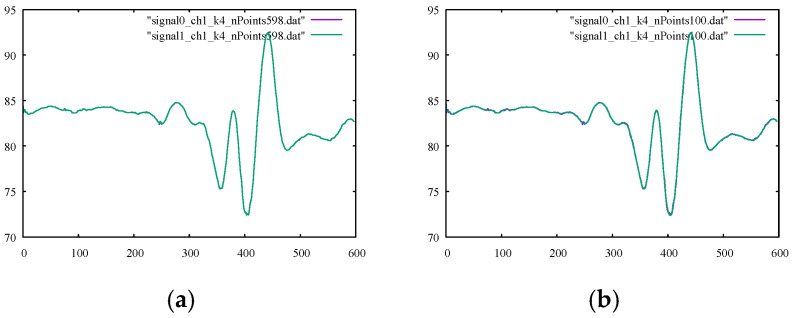

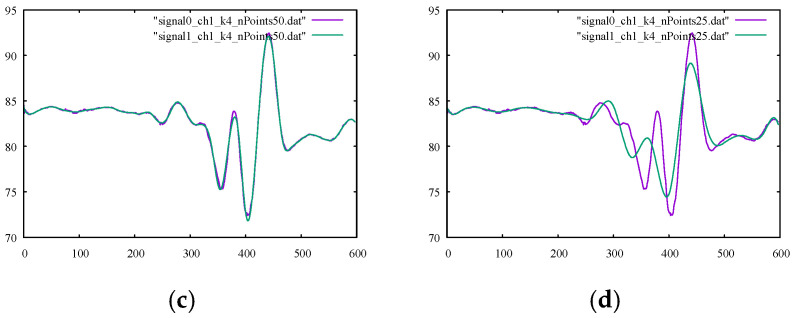
Original noisy motion signal from channel 1 of the root-joint of the kicking character versus the smoothed signal via B-spline smoothing using (**a**) 598, (**b**) 100, (**c**) 50, and (**d**) 25 control points.

**Figure 9:**
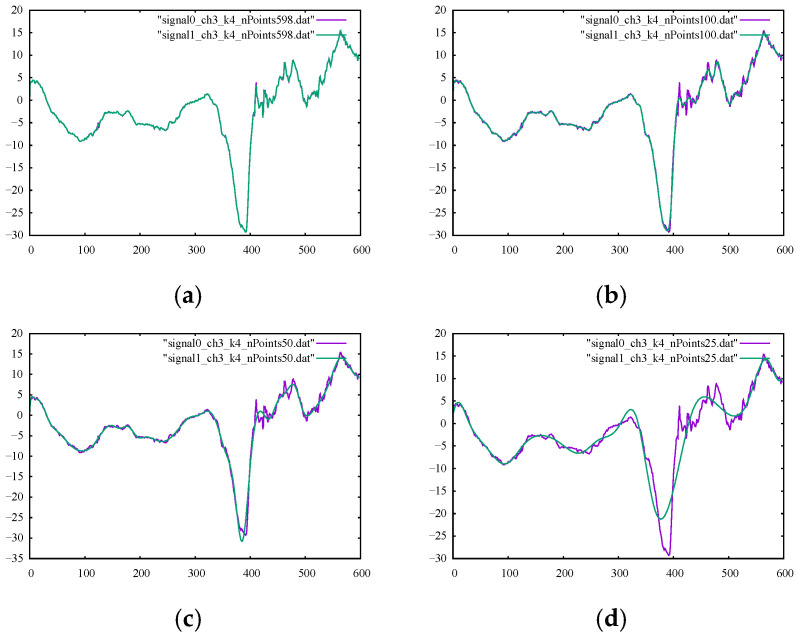
Original noisy motion signal from channel 3 of the root-joint of the kicking character versus the smoothed signal via B-spline smoothing using (**a**) 598, (**b**) 100, (**c**) 50, and (**d**) 25 control points.

The numbers provided in Table 6 for filtering using 25 control points suggested that the output signal should be checked (since the value of %SM1 was more than 30 percent) to ensure the desired level of filtering. The graphs of the filtered signals using 598, 100, 50, and 25 control points for channels 1 and 3 with their corresponding original signals are shown in Figure 8 and Figure 9, respectively. It is observable that, for both channels, the output signals using 25 control points did not conform well with the general trend of the signal curve. It is also important to note that using the same number of control points as our data points essentially led to an interpolation curve. Therefore, choosing the proper number of control points to be used with the B-spline smoother was crucial to ensure correct, smooth signal reconstruction. The proper number of control points could be found readily with few numerical experiments.

**Figure 10:**
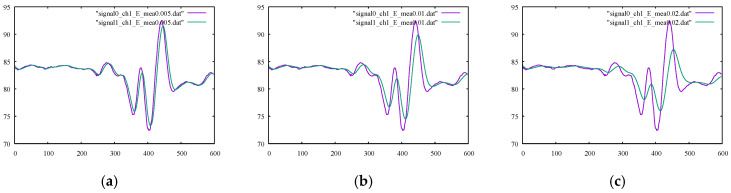
Original noisy motion signal from channel 1 of the root-joint of the kicking character versus the smoothed signal via the Kalman filter using (**a**) $E_{mea}$ = 0.005, (**b**) $E_{mea}$ = 0.01 and (**c**) $E_{mea}$ = 0.02.

**Figure 11:**
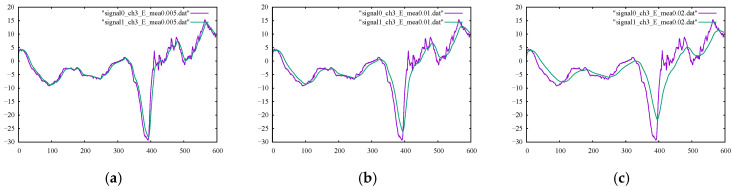
Original noisy motion signal from channel 3 of the root-joint of the kicking character versus the smoothed signal via the Kalman filter using (**a**) $E_{mea}$ = 0.005, (**b**) $E_{mea}$ = 0.01 and (**c**) $E_{mea}$ = 0.02.

Next, the Kalman filter was utilized to process the signals of the kicking character, and the values of the SM1 and SM2 measures are given in their corresponding columns in Table 4 and Table 5, respectively. Moreover, their corresponding percentages of relative difference are listed in Table 6 and Table 7. By comparing the smoothness metrics in Table 7 with the corresponding values reported for other the methods studied, the Kalman filter provided fewer smooth outputs in terms of the SM2 parameter. The SM1 percentage of relative difference values provided in Table 6 were also too high. The output signals, as well as their original signals, using various Kalman filter parameters $E_{mea}$ are shown in Figure 10 and Figure 11. Upon inspection of the filtered signals, we confirmed that only the outputs using $E_{mea}$ = 0.005 properly reproduced the desired filtering. Moreover, the filtered signals occasionally showed noisy movements because of instantaneous loops of estimated error and gain.

The results presented in this section can be set as benchmark for the considered methods, as well as for other filtering methods, when used for motion signals. In addition, the results showed how different parameters affected the outputs of the filtering methods. The B-spline filter provided the smoothest outputs and is recommended for scenarios when calculations of the derivatives of the signal are needed.

## 4. Conclusions

A comparison study for various filtering methods, namely moving average, B-spline, and Kalman filters, for motion signal smoothing was presented. The effect of different adjustable filter parameters on their smoothing performances was investigated. The B-spline smoother, in particular, showed a superior performance in achieving high-quality, smooth motion signals in the presence of low-, mid-, and high-frequency components in the signal. Moreover, the degree of continuity (smoothness) of the signal curves could be increased readily. In addition, because of the piecewise nature of B-spline curves, the method offered a great deal of flexibility. These features are essential in downstream data processing when calculations of the derivatives of a signal curve are needed, for example, in clustering and classifier training. The moving average method offered very low computational cost. However, because of highly localized computations, the filter did not perform well in the presence of high-frequency noises in the signal. The Kalman filter offered an online estimate of the true signal. The performance of the filter, however, was highly dependent on the estimated measurement error parameters provided by the user. Moreover, the smoothness of the output signal may not be sufficient for the desired applications.

## Figures and Tables

**Table 1:** Performance metrics of the moving average filtering method for different window types and sizes. The definitions of the symbols used are provided in the corresponding explanations for Equations (9)–(11). * WT: window type; ^#^ WS: window size.

* WT	^#^ WS	SM1_0_	SM2_0_	SM1_1_	SM2_1_	%SM1	%SM2
asym.	2	64.87	19.08	28.12	2.64	56.65	86.17
asym.	3	64.87	19.08	20.29	1.67	68.73	91.25
sym.	3	64.87	19.08	23.14	1.15	64.33	93.97
sym.	5	64.87	19.08	15.48	0.56	76.14	97.07

**Table 2:** Performance metrics of the B-spline smoothing method for different numbers of control points for approximation. The definitions of the symbols used are provided in the corresponding explanations for Equations (9)–(11). * No. CP: number of control points.

* No. CP	SM1_0_	SM2_0_	SM1_1_	SM2_1_	%SM1	%SM2
500	64.87	19.08	23.06	0.88	64.46	95.38
200	64.87	19.08	9.47	0.03	85.39	99.83
100	64.87	19.08	8.21	0.00	87.34	100.00
50	64.87	19.08	8.07	0.00	87.56	100.00

**Table 3:** Performance metrics of the Kalman filter using different measurement error $E_{mea}$ values.

Measurement Error	SM1_0_	SM2_0_	SM1_1_	SM2_1_	%SM1	%SM2
$E_{mea}$= 0.01	64.87	19.08	8.69	0.08	86.60	99.60
$E_{mea}$= 0.02	64.87	19.08	7.82	0.02	87.95	99.87

**Table 4:** The values of the SM1 performance metric for moving average, B-spline, and Kalman filters for signals from channels 1 and 3 of the root-joint for the kicking model using various control parameters.

			Moving	Average				B-Spline				Kalman	
		asym.	asym.	sym.	sym.	*p* = 3						$E_{mea}$	
Ch.	SM1_0_	2	3	3	5	598	200	100	50	25	0.005	0.01	0.02
1	79.42	75.57	70.95	76.04	74.61	78.85	76.24	75.37	73.82	49	66.01	53.45	36.81
3	216.45	168.4	140.58	173.06	154.78	210.78	168.62	143.76	134.84	109.41	127.76	110.64	88.94

**Table 5:** The values of the SM2 performance metric for moving average, B-spline, and Kalman filters for signals from channels 1 and 3 of the root-joint for the kicking model using various control parameters.

			Moving	Average				B-Spline				Kalman	
		asym.	asym.	sym.	sym.	*p* = 3						$E_{mea}$	
Ch.	SM2_0_	2	3	3	5	598	200	100	50	25	0.005	0.01	0.02
1	4.9	1.17	0.85	0.98	0.75	3.36	0.7	0.43	0.38	0.08	0.35	0.18	0.07
3	258.55	50	23.03	40.23	17.7	172.27	15.34	3.41	1.54	0.16	5.48	1.85	0.67

**Table 6:** The percentages of difference of the SM1 performance metric (100 × (SM1_0_ − SM1_1_)/SM1_0_) for moving average, B-spline, and Kalman filters for signals from channels 1 and 3 of the root-joint for the kicking model using various control parameters.

		Moving	Average				B-Spline				Kalman	
	asym.	asym.	sym.	sym.	*p* = 3						$E_{mea}$	
Ch.	2	3	3	5	598	200	100	50	25	0.005	0.01	0.02
1	4.86	10.67	4.26	6.06	0.72	4.01	5.11	7.06	38.3	16.88	32.71	53.65
3	22.2	35.05	20.04	28.49	2.62	22.09	33.58	37.7	49.45	40.97	48.88	58.91

**Table 7:** The percentage of difference of the SM2 performance metric (100 × (SM20 − SM21)/SM20) for moving average, B-spline, and Kalman filters for signals from channels 1 and 3 of the root-joint for the kicking model using various control parameters.

		Moving	Average				B-Spline				Kalman	
	asym.	asym.	sym.	sym.	*p* = 3						$E_{mea}$	
Ch.	2	3	3	5	598	200	100	50	25	0.005	0.01	0.02
1	76.14	82.63	79.96	84.69	31.41	85.76	91.29	92.33	98.46	92.94	96.3	98.51
3	80.66	91.09	84.44	93.15	33.37	94.07	98.68	99.4	99.94	97.88	99.29	99.74

## Data Availability

Publicly available datasets were analyzed in this study. This data can be found here: http://mocap.cs.cmu.edu.
